# Characteristics of Dye-Sensitized Solar Cell Assembled from Modified Chitosan-Based Gel Polymer Electrolytes Incorporated with Potassium Iodide

**DOI:** 10.3390/molecules25184115

**Published:** 2020-09-09

**Authors:** Aimi Mahirah Zulkifli, Nur Izzah Aqilah Mat Said, Shujahadeen Bakr Aziz, Elham Mohammed Ali Dannoun, Shameer Hisham, Shahan Shah, Amnani Abu Bakar, Zul Hazrin Zainal, Hairul Anuar Tajuddin, Jihad Mohammed Hadi, Mohamad Ali Brza, Salah Raza Saeed, Peshawa Omer Amin

**Affiliations:** 1 Visible Spectrum Laboratory, Centre for Ionics University of Malaya, Department of Physics, Faculty of Science, University of Malaya, Kuala Lumpur 50603, Malaysia; aimimahirahzulkifli@gmail.com (A.M.Z.); izzahaqilah97@gmail.com (N.I.A.M.S.); shahan.shah53@yahoo.com (S.S.); zul_hazrin@um.edu.my (Z.H.Z.); 2 Advanced Polymeric Materials Research Lab., Department of Physics, College of Science, University of Sulaimani, Qlyasan Street, Sulaimani 46001, Iraq; 3 Department of Civil Engineering, College of Engineering, Komar University of Science and Technology, Sulaimani 46001, Iraq; 4 Associate Director of General Science Department, Woman Campus, Prince Sultan University, Riyadh 11586, Saudi Arabia; elhamdannoun1977@gmail.com; 5 Organic Research Laboratory, Department of Chemistry, Faculty of Science, University of Malaya, Kuala Lumpur 50603, Malaysia; shameerh@um.edu.my (S.H.); hairul@um.edu.my (H.A.T.); 6 Department of Civil Engineering Technology, Faculty of Engineering Technology, University Tun Hussein Onn Malaysia, Edu Hub Pagoh, Pancor Johor 84600, Malaysia; amnaniabubakar@gmail.com; 7 College of Engineering, Tishk International University, Sulaymaniyah 46001, Iraq; jihad.chemist@gmail.com; 8 Department of Medical Laboratory of Science, College of Health Sciences, University of Human Development, Kurdistan Regional Government, Sulaimani 46001, Iraq; 9 Manufacturing and Materials Engineering Department, Faculty of Engineering, International Islamic University of Malaysia, Kuala Lumpur 50603, Gombak, Malaysia; mohamad.brza@gmail.com; 10 Charmo Research Center, Unit of Physics, Charmo University, Peshawa Street, Chamchamal, Sulaimani 46001, Iraq; salah.saeed@charmouniversity.org (S.R.S.); peshawa.amin@univsul.edu.iq (P.O.A.)

**Keywords:** gel polymer electrolyte, impedance study, ionic conductivity, dielectric properties, UV-VIS study, dye-sensitized solar cell (DSSC)

## Abstract

In the present work, phthaloyl chitosan (PhCh)-based gel polymer electrolytes (GPEs) were prepared using dimethylformamide (DMF) as a solvent, ethyl carbonate (EC) as a co-solvent, and a set of five quaternaries of potassium iodide (KI) as a doping salt, which is a mixed composition of iodine (I_2_). The prepared GPEs were applied to dye-sensitized solar cells (DSSC) to observe the effectiveness of the electrolyte, using mesoporous TiO_2_, which was sensitized with N3 dye as the sensitizer. The incorporation of the potassium iodide-based redox couple in a polymer electrolyte is fabricated for dye-sensitized solar cells (DSSCs). The number of compositions was based on the chemical equation, which is 1:1 for KI:I_2_. The electrical performance of prepared GPE systems have been assessed using electrical impedance spectroscopy (EIS), and dielectric permittivity. The improvement in the ionic conductivity of PhCh-based GPE was observed with the rise of salt concentration, and the maximum ionic conductivity (4.94 × 10^−2^ S cm^−1^) was achieved for the 0.0012 mol of KI:I_2_. The study of dielectric permittivity displays that ions with a high dielectric constant are associated with a high concentration of added ions. Furthermore, the gel polymer electrolyte samples were applied to DSSCs to detect the conversion effectiveness of the electrolytes. For electrolytes containing various content of KI:I_2_ the highest conversion efficiency (η%) of DSSC obtained was 3.57% with a short circuit current density (Jsc) of 20.33 mA cm^−2^, open-circuit voltage (Voc) of 0.37 V, fill factor (FF) of 0.47, as well as a conductivity of 2.08 × 10^−2^ S cm^−1^.

## 1. Introduction

In recent decades a massive increase in energy demanded and concerns with using fossil energy resources, with respect to their environmental effect and deficiency of storage, have become a global energy problem. The answer to this problem is to find a cheap, clean, and renewable energy source and alternative to fossil fuels. Solar energy is the most promising energy source among all renewable energy resources. Efficient conversion of solar energy into electric power has been a promising solution to the energy crisis. Solar cells function to capture free sunlight energy and convert it to electrical power. Currently, crystalline silicon solar cells comprise the majority of solar cells. However, high manufacturing cost and environmental issues associated with the manufacture of crystallized silicon have promoted the development of other types of solar cells. Dye-sensitized solar cells (DSSCs) are another important type of solar cells, showing potential as a next-generation photovoltaic device to replace silicon solar cells [1,2]. O’Regan and Gratzel in 1991 developed a new type of solar cell called the dye-sensitized solar cell (DSSC), because of novel properties became a new source of renewable energy [3].

An electrolyte in a DSSC is one of the components that plays an important role in DSSCs because the charge transport between the electrodes happens through it [4]. Other factors, such as sensitizer and semiconductor energetics also affect the power conversion efficiency (PCE) of DSSCs. A sensitizer governs the transportation of electrons and photon harvesting after the surface of nano-structured semiconductor is injected with electrons [5]. Additionally, the conduction-band electrons’ recombination and forming of dye accumulation on the semiconductor surface are the key reasons for the decline in conversion efficiency for many organic dyes [6]. Generally, electrolytes are classified into several types, such as liquid, quasi-solid state, and solid-state electrolytes. Due to a crucial impact on the efficiency of the solar cell, electrolytes have been subjected to extensive studies [2,4,7,8,9,10]. The liquid electrolytes are considered an important factor that affects the long lifetime because of volatilization problems related to the long-term operation of DSSC, as well as during fabrication, the handling of liquid electrolytes is quite difficult. To overcome these problems several solutions have been suggested and solid-state electrolytes control the dissolving of electrolytes and leakage problem, but they have low ionic conductivity. In addition, gel polymer electrolytes or quasi-solid state electrolytes provide an alternative for long-term operation based on preventing volatilization and corrosion with a counter electrode [11,12]. An electrolyte in a DSSC serves to restore the dye after oxidation. The three sorts of electrolytes used in DSSCs are termed liquid, quasi-solid-state, and solid-state electrolytes [11]. However, the quasi-solid electrolyte has one shortcoming, it is quite dependent on the working temperature of the solar cell, where high temperatures cause a phase transformation from a gel state to a solution state [13,14]. Gel polymer electrolytes are made by an organic liquid plasticizing solvent mixed with salt, then added to a polymer to provide mechanical stability.

High dielectric constant, high ambient ionic conductivity, mechanical flexibility (different shapes), and good electrode/electrolyte are properties that make GPEs subject to intensive study by many researchers and have become a common material used in electrochemical devices compared to liquid electrolytes or SPEs [15]. Lately, various studies in the literature have been conducted on chitosan owing to having definite properties that attract many researchers, as it offers several potentials in industrial, biomedical, and pharmaceutical applications [16,17]. Cellulose is the supreme abundant natural polymer beyond chitosan, and it is obtained from *N*-deacetylated chitin. It constitutes a host polymer for electrolytes since it can dissolve ionic salts, and is able to form a high conductivity polymeric system due to multifunctional properties and the protonated amino group in its chain structure. Using chitosan as an electrolytesis limited because it is typically insoluble in all organic solvents while it is only soluble in dilute acetic solution. Thus, to widen the range of the solubility of chitosan, the modification of chitosan to phthaloyl chitosan (PhCh) has been done as the solubility of chitosan can be enhanced by modifying it to PhCh. By replacing two-hydroxyl groups with a hydrophobic Ph group, this modification is known as phthaloylation, which decreases the crystallinity and increases the solubility of chitosan. Afterward, PhCh can be dissolving in several organic solvents, such as dimethylformamide (DMF), dimethyl sulfoxide (DMSO), and pyridine [18,19,20]. The presence of a hydrophobic Ph group in chitosan leads to destroying the hydrogen bond that is formed from the interaction between the solvents and its amino and hydroxyl groups, and it also becomes a proper host polymer in the PEs. Additionally, excluding enhancing the solubility of the polymer, numerous styles have been explored and reported by previous researchers, with the improvement of conductivity in the GPEs based on PhCh, such as PhCh:EC:PC:TPAI:LiI [3], PhCh:PEO:NH_4_I:BMII [21], and PhCh:EC:DMF:TPAI [22]. The maximum ionic conductivity for the above-mentioned system has been found to be 5.46 × 10^−3^ S/cm. In the polymer-based electrolytes, the conductivity is strongly dependent on the ionic compound which is present in the electrolyte systems. Correspondingly, the ionic compound leads to the presence of redox mediators in the PEs. Regarding this, the KI salt has been used to provide mobile ions and regenerate the oxidized electrolyte, closing the circuit, whereas the common disadvantage of using an inorganic salt is poor solubility at room temperature [23,24].

The iodide/triiodide (I^−^/I_3_) redox couple is the most popular, and it has preferred the greatest stable and effectual in DSSC. Moreover, the iodide/triiodide couple has found to be an appropriate redox potential, owing to it having enough solubility and not absorbing too much light [25]. On the other hand, cobalt (Co (II) and Co (III))-based redox electrolytes possess some outstanding characteristics, like non-volatility, safety, and being weakly colored. It has overcome the main disadvantage of the iodide/triiodide (I^−^/I_3_) redox couple due to it limiting the efficiency of the iodide/triiodide (I^−^/I_3_) redox couple. With the presence of Co (II) and Co (III), the misalliance between the redox potential of iodide/triiodide (I^−^/I_3_) and a typical sensitizer can be reduced significantly. Regarding this, several studies were reported in the literature using cobalt-based redox electrolytes for the application of DSSCs [26,27,28]. Through using I^−^/I_3_ as a redox mediator the probability of the formation of polyiodide is very high and exhibits lower conductivity of the GPE. Additionally, the amount adding of KI:I_2_ was varied and the composition is based on the chemical equation:

I^−^ + I_2_→ I_3_^−^ (1)

Thus, in this research, the correlation between the formation of polyiodide and the variation of molarity will be observed. The prepared GPEs were applied to DSSC to observe the effectiveness of the electrolyte, using mesoporous TiO_2_ sensitized with N3 dye as the sensitizer. The prepared PhCh-based GPEs have been characterized utilizing electrical impedance spectroscopy, dielectric properties, and the current density-voltage curve.

## 2. Results and Discussion

### 2.1. Study of Impedance Spectroscopy

**Figure 1:**
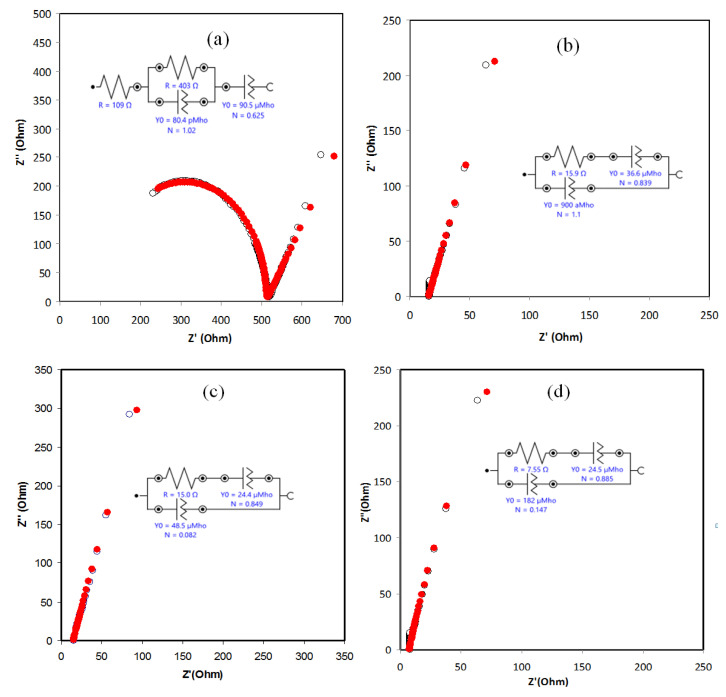

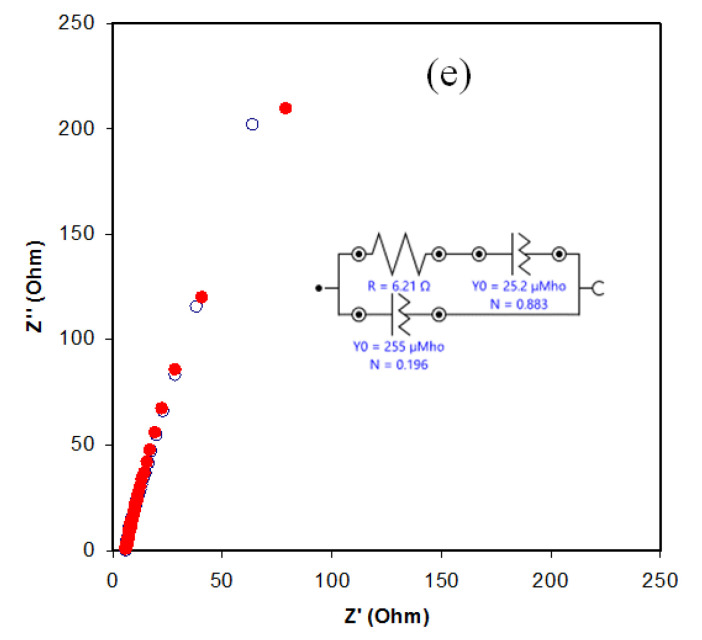
Nyquist plot (*Z_i_* versus *Z_r_*) of gel polymer electrolytes with room temperature for (**a**) I0, (**b**) I1, (**c**) I2, (**d**) I3, and (**e**) I4 systems. It is obvious that the plot for the sample of I0 displays a semi-circle due to its incorporated with no KI salt. Subsequently, the spike region increases upon adding the KI salt, and the semi-circle was disappeared at the high-frequency regions for the samples of I1–I5.

To understand the ionic transport and electrical properties in the polymer-based electrolytes, the analysis of electrical impedance spectroscopy (EIS) is an outstanding informative method [29]. The EIS was performed in this study to characterize the impedance plots for the prepared gel polymer electrolyte GPE systems. This is done by applying a small oscillatory voltage to the sample and measuring the resulting oscillatory current through the sample. The phase and amplitude difference between the voltage across, and the current through, the sample determine the impedance of the sample at that frequency [15]. Figure 1 explains the impedance spectra of Nyquist plots (*Zi* versus *Zr*) of PhCh-based GPE for the samples of I0, I1, I2, I3, and I4 incorporated with 0, 0.0003, 0.0006, 0.0009, and 0.0012 mol of KI/I_2_, respectively, at room temperature. The salt influence on ionic conductivity of the GPE-incorporated phthaloyl chitosan has been carried out by adding varying mole ratios of KI/I_2_. Overall, the Nyquist plots show a half semicircle for the sample without KI salt. This is attributed to the bulk resistance with the charge transfer resistance representing the diffusion resistance of electrolytes. However, the tails (i.e., spike) are obtained by adding salt, which corresponded to the blocking effect of the electrode [30,31,32]. Additionally, the bulk electrical resistance *R_b_* value for each GPE system has been determined from the complex impedance plots at the low-frequency intercept on the real axis (*Z_r_*). Obviously, the system with the minimum *R_b_* value has the maximum DC ionic conductivity. It can be perceived that, by increasing the salt concentration, the bulk resistance is reduced, and the ionic conductivity is improved owing to the rise in the number of mobile ions in the gel electrolytes and the rise of salt dissociation which leads to the increase of conductivity. The DC conductivity was calculated for the all of the electrolyte systems by utilizing the Equation (2) [33,34,35].

(2) $$\sigma \;=\;\frac{t}{R_{B}\times A}$$

**Figure 2:**
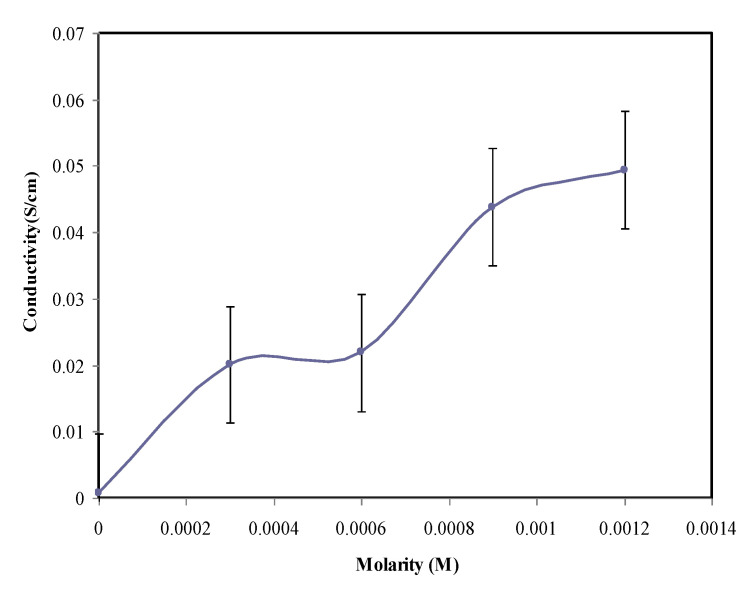
Conductivity versus molarity of KI salt.

The variation of conductivity against the molarity of the KI/I_2_ ratios for the GPE samples is presented in Figure 2. The error bars are also shown in Figure 2 in which three samples were used to calculate the error bars. The optimum value of ionic conductivity was found to be 4.94 × 10^−2^ S cm^−1^, achieved from the sample that includes the highest amount (0.012 mol) of added salt at room temperature, as shown in Table 1, which indicates the highest ion mobility among the GPE systems. It can be noticed that the conductivity increases with the increment of the salt concentration. This suggests that the ion migration in the gel polymer electrolyte samples integrated with KI salt was signified by a resistor [36]. In comparison, the room temperature ionic conductivity of the present study is fairly near to some other former studies based on PhCh; for instance, the conductivity obtained for the PhCh-PEO-TPAI-EC system was 1.11 × 10^−2^ S cm^−1^ examined by Buraidah [37]. Our previous work consisted of PhCh reacted with the KSeCN salt PhCh:EC-DMF:KSeCN system, with a value of 4.75 × 10^−2^ S cm^−1^ [38]. Consequently, for the PVA:EC:PC:KI system is was 1.25 × 10^−2^ S cm^−1^, as reported by Aziz [39].

The formation of poly-iodide is formed when excess iodide reacts with tri-iodide as shown in the equation below:

I_3_^−^ + I^−^→I_4_^−2^ (3)

**Figure 3:**
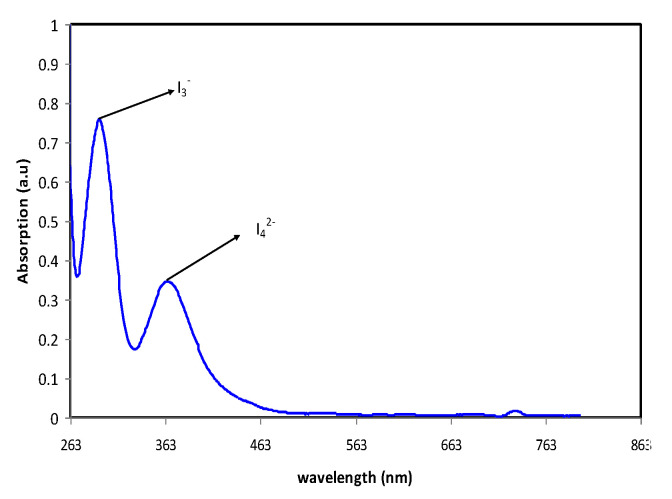
The peak of wavelength for polyiodide for sample I_3_.

UV-VIS spectroscopy is the most vital technique to comprehend the transition of electrons. The region in which the electrons hopped from the ground energy state to the upper energy state by an incident photon is called the absorption edge. In the amorphous materials, the bandgap energy can be determined from the absorption edge. To confirm the formation of the polyiodide, ultraviolet visible (UV) spectroscopy has been used [40]. Figure 3 below displays the graph of absorbance against wavelength for sample I3. The figure displayed two surface plasma resonance (SPR) peaks at 288 nm and 363 nm, indicating the presence of I_3_^−^ and I_4_^2−^ ions, respectively. This phenomenon will happen when the natural frequency of surface electrons and the photon frequency are equal to each other [41]. The peak of the wavelength at 288 nm indicates the presence of I_3_^−^ ions [42], while for the presence of I_4_^2−^ (polyiodide) it is detected at the peak at 359 nm [43]. The peaks obtained are approximate to the value of I_3_^−^ ions and I_4_^2−^ ions which are 298 nm and 366 nm, respectively.

### 2.2. Dielectric Studies

In this study, the capacity of the dielectric GPE based on PhCh has been characterized to determine the material’s ability to attain the electrical storage charge through employing dielectric permittivity. Additionally, it can estimate the ion transportation phenomena that occurred in the electrolyte samples. The dielectric permittivity consists of two components that are a dielectric constant and dielectric loss. The dielectric constant (ε′) signifies the capability of materials to collect the electric charge, and the dielectric loss (ε″) represents the loss of energy to ion mobility and aligns dipoles once the electric field polarity reverses quickly, and is typically associated with ionic transport. The dielectric constant and dielectric loss are designated as a real part of permittivity (ε′) and the imaginary part of permittivity (ε″), respectively. The formula for each permittivity was shown in Equations (4) and (5) [44,45].

(4) $$\varepsilon^{\prime }\;=\;\frac{t}{\omega A\varepsilon_{o}}\left[\frac{Z^{\prime \prime }}{{\left(Z^{\prime }\right)}^{2}+{\left(Z^{\prime \prime }\right)}^{2}}\right]$$

(5) $$\varepsilon^{\prime \prime }\;=\;\frac{t}{\omega A\varepsilon_{o}}\left[\frac{Z^{\prime }}{{\left(Z^{\prime }\right)}^{2}+{\left(Z^{\prime \prime }\right)}^{2}}\right]$$

**Figure 4:**
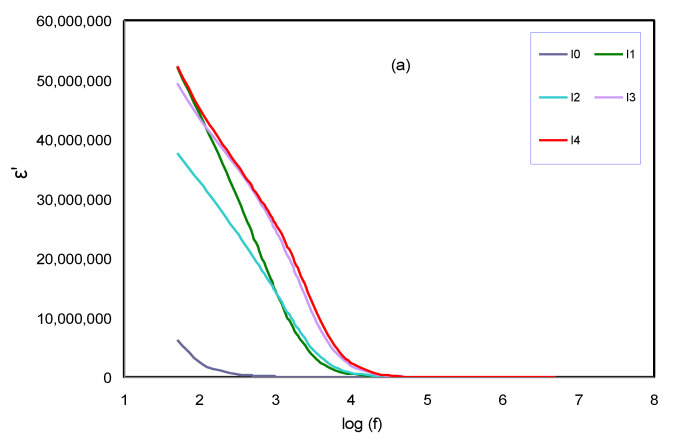

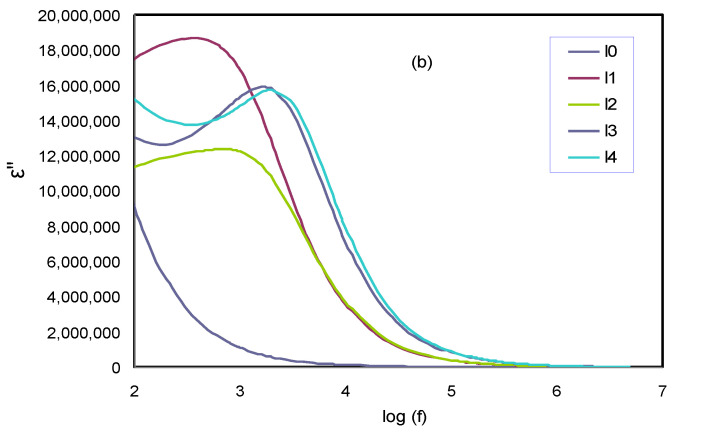
(**a**). Variation of dielectric constant (ε′) with frequency dependent of GPE based on I0, I1, I2, I3, and I4 systems at ambient temperature; (**b**). Variation of dielectric loss (ε″) with frequency dependent of GPE based on I0, I1, I2, I3, and I4 systems at ambient temperature.

Figure 4a demonstrates the dielectric constant versus frequency at room temperature for the prepared GPE samples. The Sharpe surge in the real dielectric constant for the samples indicate the higher ionic conductor at a low frequency associated with the polarization effect due to the accumulation of ion species near the blocking electrode. Since the dielectric constant is high, this leads to high storage of the dipole electric charge per unit volume [46]. In the meantime, the dielectric constant value reaches its lowest value and gets close to zero at high frequency, owing to the dominance of the relaxation process occurring, and lowers the dielectric constant. This occurred because of the quick change in the direction of the field, which results in inadequate time for the ions to accumulate at the electrode [47,48,49]. Beyond that, the increase of the dielectric constant with the increasing molarity of the salt can be observed. Among the GPE systems, the highest value of the dielectric constant (i.e., ε′) was recorded for the maximum content of KI added salt. This is because the interaction between the polar molecules with the ions from the salts helps in the dissociation of ions and increases the concentration of ion species in the polymer matrix [50]. By means of the graph of dielectric loss (ε″) in Figure 4b, the same behavior can be observed as the dielectric constant which shows the variation of dielectric loss versus frequency for the whole samples at ambient temperature. One can see that, as the frequency is increased, the value of the dielectric loss decreased. The number of ion species governs the polarization of the space charge with an increase in the salt ratio, and leads to the enhancement of the accumulated space charge polarization from the increase of charge carriers by adding a greater salt ratio [51,52]. The values of dielectric constant at low frequency and ionic conductivity are shown in Table 2. It is noticeable from Table 2 that high dielectric constant corresponds to high ion conductivity.

### 2.3. J-V Characteristic of DSSC

**Figure 5:**
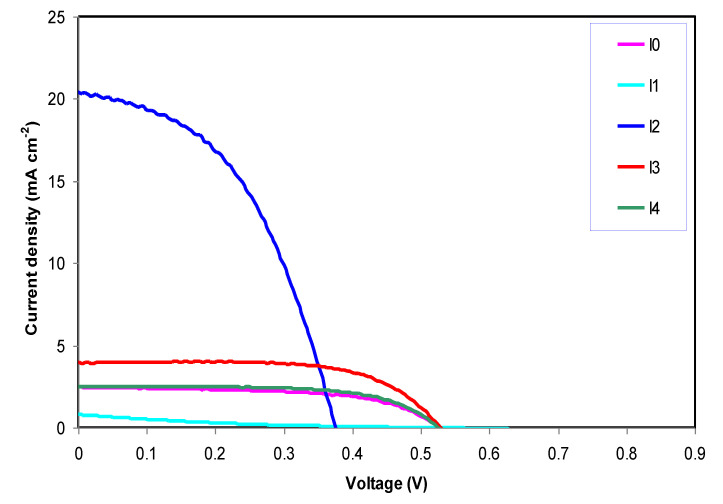
The J-V curve for the PhCh based GPE based on I0, I1, I2, I3, and I4 systems.

All the components in DSSCs have important impacts on the efficiency and stability, first, enhancing electrical conductivity and light transmittance are two properties required for a conducting oxide glass substrate. Second, porosity and morphology of the TiO_2_ surface and the amount of dye molecules absorbed on it provide a massive area of reaction sites for the absorption of incident lights [53]. Third, iodine and tri-iodide as a liquid electrolyte used in redox mediators and due to its volatilization problem of liquid electrolytes other solid state and quasi-solid electrolytes used to overcome these disadvantages [14]. The full five GPE systems prepared in this study have been approved in dye-sensitized solar cells (DSSCs). The photocurrent-photo-voltage (J-V) graph of the DSSCs was performed to illustrate the PhCh-based gel polymer electrolyte samples incorporated with 0 mol, 0.0003 mol, 0.0006 mol, 0.0009 mol, and 0.0012 mol of KI salt at room temperature, which are presented in Figure 5. Consequently, the photo-voltage characteristics shown in Figure 5 are epitomized in Table 3. The dye-sensitized solar cells prepared with the GPEs exhibit similar or higher open-circuit voltage (Voc) and lower short circuit density (Jsc) than the DSSCs with the liquid polymer electrolyte [54,55]. Several factors affect the DSSC application, such as the charge density and mobility of the ions in the polymer-based electrolytes. Through the iodide ions (I^−^), the sensitizer generates the electrons and then transportation will occur from the photo-anode to the counter electrode (CE), then rebound back to the anode. The oxidized photo-sensitizer becomes regenerated again once the electrons from the iodide ions reach the holes. When the electron transfers by the iodide ions to the sensitizer, the electrons are converts to the tri-iodide (I_3_^−^) ion and spreads to the counter electrode [56]. The parameters of solar cells and the values of the short circuit density (Jsc), open-circuit voltage (Voc), fill factor (FF), and conversion efficiency (η%) are presented in Table 3, which are obtained from the J-V curve. It can be prominent that all solar cell parameters are enhanced except Voc by incorporating 0.0003 mol of KI salt at room temperature into the PhCh-based gel polymer electrolyte sample (I1) although the extra increase of KI/I_2_ into GPE strongly influenced these parameters. Section 3.1 and 3.2 display that, with increasing KI salts into the GPE, the DC conductivity and dielectric constant are increased [57]. However, these enhancements have not to be seen in the DSSC system due to numerous issues. First, in the sample of I1, increasing Jsc to its maximum value represents more free carriers transferred from the electrolyte to the photo-anode, but Voc decreased to its minimum value. The reason for these changes is explained by the downshift of the conduction band and the Fermi level of TiO_2_ more toward the redox potential because of the increasing number of free ions in GPE and more free cations will be adsorbed and accumulated on the TiO_2_ mesoporous surface [57,58,59]. It is noticeable that the value of the DSSC efficiency was significantly enhanced from 0.06% to 3.57% up on adding of 0.0003 mol of KI/I_2_. Second, in other samples (I2, I3, and I4) open-circuit voltage increases and becomes constant, while the short circuit current density and efficiency dropped. By further increasing the KI/I2 concentration due to the aggregation of KI salt the cations do not absorb on the TiO_2_ surface and the downshift of the conduction band and Fermi level is less [60,61]. Thus, the value of (Voc) changed from 0.37 V to 0.52 V. Furthermore, the decrease of efficiency is due to the increased of I^−^ ion concentration, which contributes to ion-pairing in its place of dissociation of ions [62,63,64].

Table 4 gives information about the calculated efficiency (η%) for some systems reported in the literature that include binary salts [65,66,67,68,69,70]. In comparison, the value of efficiency (η%) of this work is close to some of these reports. Meanwhile, the reason why the efficiency for KI is higher compared to other salts may be due to the effect of lattice energy or mixed cation salt. The size of the I^−^ ion is much smaller, making it diffuse faster and the larger cation acts to produce a higher number of redox mediators [66]. The efficiency of the current work (3.57%) is of excessive significance paralleled to our previous work (2.28%) in which KSCN salt was used as a doping salt [39]. In our earlier work, KSCN is used as the ionic source. KSCN has a lattice energy (*U_L_*) of 616 kJ/mol, which is slightly lower than that estimated for KI, which is about 649 kJ/mol) [71]. Salt with lower *U_L_* tends to associate easier compared with higher *U_L_*. However, due to the lower value of *U_L_*, there is a greater chance for the cation and anion to associate again. Consequently, few free ions are available, thus resulting in a lower efficiency value. It can be seen that the I_2_ system exhibit higher efficiency compared to other systems. High concentration of ions may result in more ion association and ion triplets, thus, medium salt concentration may be better for DSSC fabrication.

## 3. Experimental

### 3.1. Modification of Chitosan to PhCh

The PhCh was attained using 1 g of low molecular weight chitosan, 30 mL of dimethylformamide (DMF), and 4.3 g of phthalic anhydride which were mixed in a round-bottomed flask. All fresh materials are acquired from Sigma-Aldrich (Darmstadt, Germany). The mixture was stimulated constantly using a magnetic stirrer under nitrogen gas (N_2_), and heated at 120 °C for 6–7 h. After that, the mixture was permitted to cool down to 60 °C and the gas pipe that connects the nitrogen gas from the condenser was taken out, the product being left overnight. Then, ice water was used to form the precipitate and filtered out using a filtration technique. Finally, ethanol was used to wash the collected precipitate in a Soxhlet extractor for 6 h. Consequently, the final product was dried and vacuumed at 60 °C in a vacuum oven for several hours.

### 3.2. Gel Polymer Electrolyte (GPE) Preparation

To make an iodide/triiodide redox couple, 0.2 g of PhCh was dissolved in 0.6 g of dimethyl formamide DMF. The mixture was heated at around 80 °C and stirred with the magnetic stirrer for a half hour until the mixture becomes homogenous. Then, under the same condition, 0.6 g of ethylene carbonate (EC) was added into the mixture and stirred constantly. After this, various levels of potassium iodide (KI) were added into the solution mixture and heated to about 80 °C for five hours. Lastly, the iodine was added to the mixture once the mixture already turned into a gelatin form, and stirred for 2 h at room temperature until the homogenous behavior was obtained. The designation and GPE compositions are summarized in Table 5.

### 3.3. Characterization of GPEs

Electrochemical impedance spectroscopy (EIS) has been used to evaluate the ionic conductivity and dielectric mechanism of the GPEs. An LCR meter (HIOKI 3531 Z Hi-tester, Hioki, Nagano, Japan) instrument was employed at a frequency range between 50 Hz and 5 MHz. For the conductivity study, the value of the ionic conductivity (σ) based on the bulk resistance from the Nyquist plot was determined using Equation (2). where *t* is the thickness of the electrolyte, *R_B_* is the bulk resistance and *A* is the cross-sectional area of the electrolyte. Additionally, both real and imaginary parts of complex permittivity (ε*) were calculated from the impedance data (i.e., Z′ and Z″). The values of the dielectric constant, ε′ and dielectric loss, ε″ were obtained utilizing the Equations (4) and (5).

### 3.4. Fabrication of Dye-Sensitized Solar Cell (DSSC)

To construct the DSSCs, fluorine tin oxide (FTO) was washed and cleaned using ethanol and distilled water (D.W.) and was used as a glass substrate. Similarly, to make a photo-anode on a conducting glass substrate, double layers of TiO_2_ were used. To prepare a first layer, 0.5 g of TiO_2_ powder was ground for a half hour in 2 mL of (HNO_3_) nitric acid using a mortar and dried in air for another 30 min, then it was sintered for half an hour at 450 °C. As for the second layer, the TiO_2_ colloidal suspension was prepared by grinding another 0.5 g of TiO_2_ powder with 2 mL of nitric acid. Correspondingly, 0.1 g of carbon wax with some drops of Triton X-100 were increased to the system and sintered in the furnace at 450 °C for an hour. After that, the electrode was cooled down to 60 °C and saturated into an ethanolic N3 dye solution for one day. After cooling, the prepared GPEs were cast into the sensitized TiO_2_ photo electrode and then sandwiched with a platinum-coated electrode. Through using an AUTOLAB electrometer, the photovoltaic performance of the DSSCs was observed. For this purpose the photocurrent density (*J*) and voltage (*V*) were recorded using the Metrohm Autolab Potentiostat/Galvanostat instrument (PGSTAT 128N, Neware, Shenzhen, China) under an irradiation intensity of 1000 W m^−2^. The area of the DSSC was 0.20 cm^2^.

## 4. Conclusions

In conclusion, PhCh as a host polymer was used to prepare a series of gel polymer electrolytes utilizing DMF as a solvent, EC as a plasticizer, and KI salt dopant, which optimized to regenerative photoelectrochemical cells. Through impedance spectroscopy and electrical equivalent circuits the bulk electrical resistance *R_b_* value for each GPE system has been determined. The highest room temperature conductivity accomplished in this project is 4.94 × 10^−2^ S cm^−1^ with 0.0012 mol of the KI/I_2_ composition. Additionally, the electrolyte sample incorporated with the highest salt content has found to be a high intensity of dielectric constant. The DSSCs have been fabricated for the entire systems and an efficiency (η%) for the N3 dye of 3.57% was obtained for a sample containing 0.0003 mol of KI salt with Jsc = 20.33 mA cm^−2^. Several factors affect the conductivity and conversion efficiency such as poly-iodide formation, lattice energy, and size of the anion. The resulting outcome in this work shows the phthaloyl chitosan-based gel polymer electrolytes have potential in the application of electrochemical devices.

## Figures and Tables

**Table 1:** The conductivity value of gel polymer electrolytes with variation molarity at ambient temperature.

Sample	Conductivity, S/cm
I0	8.213 × 10^−4^
I1	2.01 × 10^−2^
I2	2.19 × 10^−2^
I3	4.38 × 10^−2^
I4	4.94 × 10^−2^

**Table 2:** The values of ionic conductivity in direct current and dielectric constant for the all GPE systems with various molarity of KI/I_2_.

Sample Code	Ionic Conductivity σ (S/cm)	Dielectric Constant
I0	8.22 × 10^−4^	2.96 × 10^6^
I1	2.08 × 10^−2^	2.90 × 10^6^
I2	2.21 × 10^−2^	6.40 × 10^6^
I3	4.38 × 10^−2^	6.75 × 10^6^
I4	4.94 × 10^−2^	7.50 × 10^6^

**Table 3:** DSSC parameters of the GPEs based on PhCh with various molarity of KI salt.

Sample	J_sc_	V_oc_ (V)	FF	ƞ%
	(mA cm^−2^)			
I0	2.46	0.51	0.14	0.76
I1	0.78	0.48	0.47	0.06
I2	20.33	0.37	0.65	3.57
I3	3.96	0.52	0.64	1.35
I4	2.51	0.52	0.59	0.84

**Table 4:** Various GPE systems based on efficiency (η%) for the DSSCs.

GPE Systems	Sensitizer	Efficiency (ƞ%)	References
PhCh:PEO:EC:DMF:TPAI/I2	Anthocyanin	0.56	[67]
PVA:EC-PC:KI-TPAI/I2	N3	3.27	[68]
PAN:EC:PC:TPAI/I2	Chlorophyll	1.97	[69]
PEO:EC-PC-DMC:NaI/I2	N719	3.6	[70]
PAN:EC:TPAI/I2	N3	3.45	[60]
PMMA:PVDF:KI:I2:EC/PC	N719	4	[71]
PVDF:PMMA:EC: Imidazole:KI/I2	N719	3.04	[29]
PhCh:EC:DMF:KI/I2	N3	3.57	This work

**Table 5:** Designation and composition of PhCh:DMF:EC:xKI/I_2_GPE systems.

Samples	PhCh (g)	DMF (g)	EC (g)	KI/mol	I_2_/mol
I0	0.2	0.6	0.6	0.0000	0.0000
I1	0.2	0.6	0.6	0.0003	0.0003
I2	0.2	0.6	0.6	0.0006	0.0006
I3	0.2	0.6	0.6	0.0009	0.0009
I4	0.2	0.6	0.6	0.0012	0.0012
